# Effective connectivity of the anterior hippocampus predicts recollection confidence during natural memory retrieval

**DOI:** 10.1038/s41467-018-07325-4

**Published:** 2018-11-19

**Authors:** Yudan Ren, Vinh T. Nguyen, Saurabh Sonkusare, Jinglei Lv, Tianji Pang, Lei Guo, Simon B. Eickhoff, Michael Breakspear, Christine C. Guo

**Affiliations:** 1 School of Automation, Northwestern Polytechnical University, 710072 Xi’an, China; 2 QIMR Berghofer Medical Research Institute, Brisbane, 4006 Australia; 3 School of Medicine, The University of Queensland, Brisbane, 4072 Australia; 4 Institute of Systems Neuroscience, Medical Faculty, Heinrich Heine University Düsseldorf, 40225 Düsseldorf, Germany; 5 Institute of Neuroscience and Medicine, Brain & Behaviour (INM-7), Research Centre Jülich, Jülich, 52425 Germany

## Abstract

Human interactions with the world are influenced by memories of recent events. This effect, often triggered by perceptual cues, occurs naturally and without conscious effort. However, the neuroscience of involuntary memory in a dynamic milieu has received much less attention than the mechanisms of voluntary retrieval with deliberate purpose. Here, we investigate the neural processes driven by naturalistic cues that relate to, and presumably trigger the retrieval of recent experiences. Viewing the continuation of recently viewed clips evokes greater bilateral activation in anterior hippocampus, precuneus and angular gyrus than naïve clips. While these regions manifest reciprocal connectivity, continued viewing specifically modulates the effective connectivity from the anterior hippocampus to the precuneus. The strength of this modulation predicts participants’ confidence in later voluntary recall of news details. Our study reveals network mechanisms of dynamic, involuntary memory retrieval and its relevance to metacognition in a rich context resembling everyday life.

## Abstract (web summary)

While memory is often studied using voluntary recollection, the neural correlates of involuntary memory recall and its effect on cognition are unclear. Here, Ren and colleagues show that the effective connectivity from the anterior hippocampus to the precuneus can predict the strength of involuntary retrieval of episodic memory.

## Introduction

Our interactions with the world, from perception to action, are continually informed and shaped by the retrieval of memories of recent and past experiences. This information retrieval occurs naturally, without the mental effort of search, and implicitly guides behaviours. Involuntary memory retrieval typically encapsulates specific temporal and spatial information. For example, when watching a TV series, one implicitly relies upon previously viewed episodes and recalls specifics following commercial breaks. Such recall is essential to engaging in the present narrative. Involuntary retrieval of episodic memory pervades everyday life, and may indeed be more frequent than its voluntary counterpart^1,2^. Moreover, malfunction of involuntary retrieval is distressing and disabling, and may contribute to neuropsychiatric conditions, such as posttraumatic stress disorder^1,3^. Thus, elucidating the neural substrate of natural, involuntary memory retrieval has significant fundamental and clinical implications.

Current cognitive theory of episodic memory retrieval mostly depends on voluntary recollection of abstract and static stimuli, specifically the contrast between correct recall of old vs. novel objects^4–6^. Using such task designs, lesion and neuroimaging studies have demonstrated that the hippocampus is crucial for episodic memory—although the precise anatomical subdivision remains a matter of debate^7^. Neuroimaging studies have also revealed additional brain regions within the default mode network (DMN)—including precuneus, angular gyrus and medial prefrontal cortex—that are also engaged during episodic memory retrieval^8–10^. However, because lesions in these DMN regions do not typically result in memory impairment, there remains substantial debate over their functional relevance in episodic retrieval^9–14^. Intriguingly, some recent evidence has suggested that the involvement of DMN during memory retrieval might be more related to the metacognitive monitoring of retrieval success rather than the baseline performance per se^11,15–17^. This is consistent with the proposed role of DMN in introspection, self-referential processing, internal monitoring^18^, as well as broader metacognitive awareness of one’s own thinking^15,16^.

While this body of work has been highly informative, it rests upon research designs that do not embody the multimodal, dynamic and often involuntary characteristics of real-life memory. To improve the ecological validity of experimental paradigms, recent studies have thus employed naturalistic stimuli to engage and investigate neural processes underlying episodic memory retrieval^19,20^. These naturalistic functional magnetic resonance imaging (fMRI) studies, using film and spoken narratives, have further extended our understanding of the role of hippocampus to real-life, dynamic memory processing—interacting with the DMN to support episodic memory retrieval. However, what they gain in ecological validity, naturalistic paradigms typically lose to experimental constraint and the lack of control conditions. This limits the direct functional inference of brain activations. Furthermore, few studies have investigated the network mechanisms of the real-life, involuntary memory retrieval and its behavioural relevance.

To bridge this gap, we designed a paradigm based on news clips: viewing news clips is an almost daily exercise for most people, and implicitly relies upon the involuntary retrieval of information to resume viewing experience following commercial and other breaks in the narrative. Our fMRI paradigm was designed to simulate this real-life scenario in the scanner. Participants first viewed the first half of 9 news items. After a short delay with distraction, the participants then viewed the second half of 18 news clips—9 of which were a continuation of the previously viewed items, and 9 naïve (control) clips (Supplementary Figure 1a, c). We hypothesised that continued viewing of the second-half news clips would likely trigger memory retrieval of the previously viewed contents, compared to the viewing of naïve news clips. We employed general linear model (GLM) and dynamic causal modelling (DCM) to examine the network of retrieval-related brain regions and infer their effective interactions. We further investigated how network connectivity during involuntary retrieval might predict memory accuracy and confidence during delayed recall (Supplementary Figure 1e). Our study hence addresses the neural mechanism underlying dynamic, involuntary memory retrieval in a naturalistic context resembling the function of memory in real-life.

## Results

### GLM analysis of news viewing

**Fig. 1:**
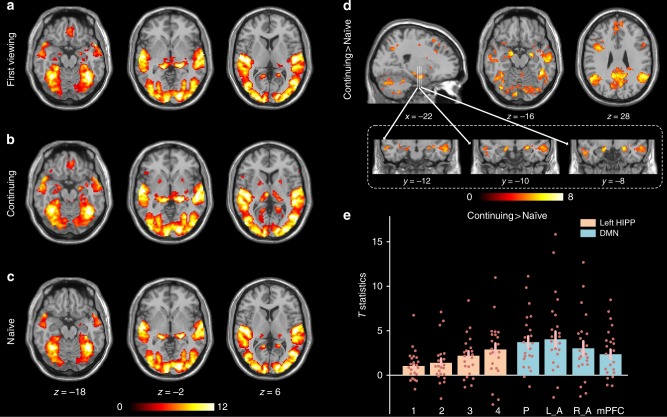
GLM results of news clip viewing. **a** Brain regions with statistically significant activation during first viewing, **b** continued-viewing, **c** naïve viewing. **d** Brain regions with greater activation to the continuing viewing than naïve viewing. **e** Average *T* statistics in left hippocampus (1–4 represents four hippocampal parcels from posterior to anterior hippocampus; see Methods, Supplementary Figure 14) and DMN (P: precuneus, L_A: left angular gyrus, R_A: right angular gyrus, mPFC: medial prefrontal cortex) across participants. Significant voxels were identified using a FWE cluster-corrected threshold *p* < 0.001, with a voxel height defined with *p* < 0.001. Error bars signify SEM

All news viewing conditions activated primary and association visual cortices, primary and association auditory cortices, fusiform gyrus, hippocampus, precuneus, Broca’s area, dorsolateral prefrontal cortex, angular gyrus, supramarginal gyrus and premotor cortex (Fig. 1a–c, *p* < 0.001 FWE corrected), showing similar pattern to the ISC maps. Compared to the naïve viewing condition, continued-viewing evoked stronger activation in the anterior hippocampus, precuneus, angular gyrus, superior medial frontal cortex, superior and middle temporal gyrus, supramarginal gyrus, dorsolateral prefrontal cortex, premotor cortex, secondary visual cortex and Crus I/II (Fig. 1d, *p* < 0.001 FWE corrected). Voxel-wise results revealed robust activations in voxels in the anterior hippocampus but not in the middle and posterior hippocampus (Fig. 1d). We then segmented the whole hippocampus into anterior to posterior parcels. This revealed a distinct anterior-to-posterior gradient in the hippocampus, with the strongest activation in the anterior hippocampus, systematically weakening towards the posterior portions (Fig. 1e). In addition, the left hippocampus appeared to be more engaged than the right hippocampus in both cluster extent and activation strength (Fig. 1d). Medial prefrontal cortex evoked the weakest activation, consistent with a previous study on involuntary memory that showed little involvement from prefrontal regions^21^. Note that we did not detect any regions showing greater functional activation during the naïve than the continued-viewing condition at this threshold. Participants also completed an event-related face-name association task (Supplementary Figure 2b, d). The news viewing paradigm evoked slightly stronger activations in the core network underlying episodic memory retrieval, including the hippocampus, precuneus, angular gyrus and medial prefrontal cortex than this conventional task (Supplementary Figure 3 and Supplementary Note 1).

### Effective connectivity network of episodic memory retrieval

To investigate the network mechanisms underlying these effects, we next employed DCM to infer effective connectivity of retrieval-related regions during continuing viewing. We selected four regions revealed by the continuing > naïve contrast: the anterior hippocampus, angular gyrus, precuneus—which are all frequently implicated in the episodic memory retrieval^10^—and the secondary visual cortex—to model the sensory input. The relationship with other regions revealed by this contrast, such as superior medial frontal cortex, dorsolateral prefrontal cortex, premotor cortex, Crus I/II, etc., could be important for understanding the influence of attention network and the cerebellum on episodic memory and may be explored in future studies. In addition, we constrained our model to the left hemisphere, due to the stronger hippocampal and angular gyrus activations in comparison to the right hemisphere (Fig. 1d, e).

**Fig. 2:**
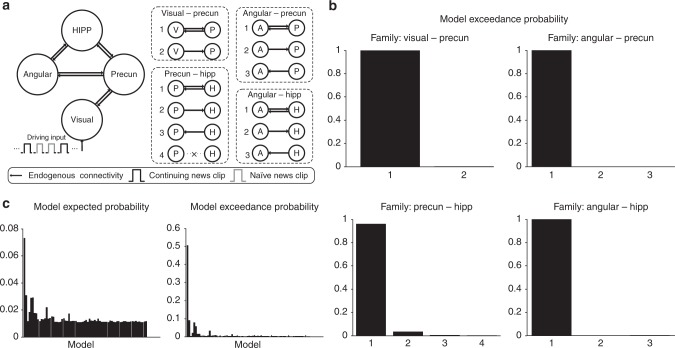
DCM analyses of endogenous connectivity for second viewing. **a** Specification of the model space in terms of four sets of connectivity families, including connections between visual and precuneus, angular gyrus and precuneus, precuneus and hippocampus, angular gyrus and hippocampus, resulting in 72 competing models. For all the models, the visual ROI receives the driving input. **b** Exceedance probability for the DCM model families in the BMS, including visual–precuneus families, angular gyrus–precuneus families, precuneus–hippocampus families, and angular gyrus–hippocampus families. The label of horizontal axis for each box corresponds to the label in (**a**). Bidirectional families are given first in each box. **c** Results of the BMS for all the 72 models. HIPP = Hippocampus; Precun = Precuneus; Angular = Angular gyrus

We first established the endogenous effective connectivity between these ROIs (see Methods). We specified model space in terms of four types of connectivity families (Fig. 2a). Combinations of these four types of families resulted in 72 models. Bayesian model comparison (BMS) with random effects analysis was first performed on each of the families individually (Fig. 2b), which allows us to focus on the existence and direction of specific connections within each family. The BMS of all the four connectivity families favours bidirectional connections (the first choice in each family), with the model exceedance probabilities exceeding 0.95 for all families with bidirectional connectivity (Fig. 2b). The BMS procedure was then performed on all 72 models. The overall winning model is the one which combines each winning family (Supplementary Figure 4), hence with all connections bidirectional, with an exceedance probability of 0.51 (Fig. 2c).

Notably, data from the corresponding ROIs in the right hemisphere generated the same winning model as the left hemisphere (Supplementary Figure 5).

**Fig. 3:**
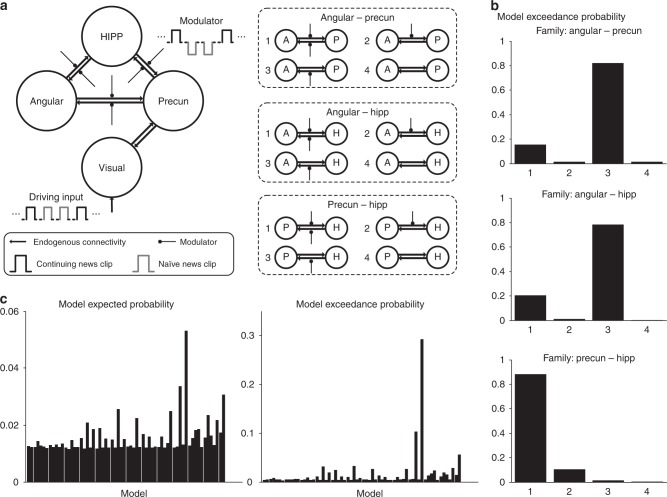
DCM analyses of modulations for the second news viewing. The models are specified by adding modulations corresponding to the continued-viewing effect. **a** Identifications of the model space in terms of three sets of modulator families, including modulator acting on connections between angular gyrus and precuneus, angular gyrus and hippocampus, precuneus and hippocampus, resulting in 64 competing models. **b** Exceedance probability (right) for the DCM model families in the BMS, including angular gyrus–precuneus families, and angular gyrus–hippocampus families, precuneus–hippocampus families. The label of horizontal axis for each box corresponds to the label in (**a**). **c** Results of the BMS across all the 64 models. HIPP = Hippocampus; Precun = Precuneus; Angular = Angular gyrus

We then investigated the effects of modulation by involuntary retrieval (that is, informed by the continuing > naïve contrast), building on this intrinsic architecture by introducing a contextual modulation. Three modulator families were constructed to by adding modulators to the endogenous connections between the retrieval-related ROIs (Fig. 3a). Each connection between angular gyrus, precuneus and hippocampus can be either modulated or not, resulting in four possible models for three families respectively and 64 models in total. The application of BMS to these three modulator families (Fig. 3b) respectively, selected families with modulators acting on the connections from the precuneus to the angular gyrus (exceedance probability = 0.82), from the hippocampus to the angular gyrus (exceedance probability = 0.78), and modulatory effects on the bidirectional connections between the precuneus and the hippocampus (exceedance probability = 0.88).

**Fig. 4:**
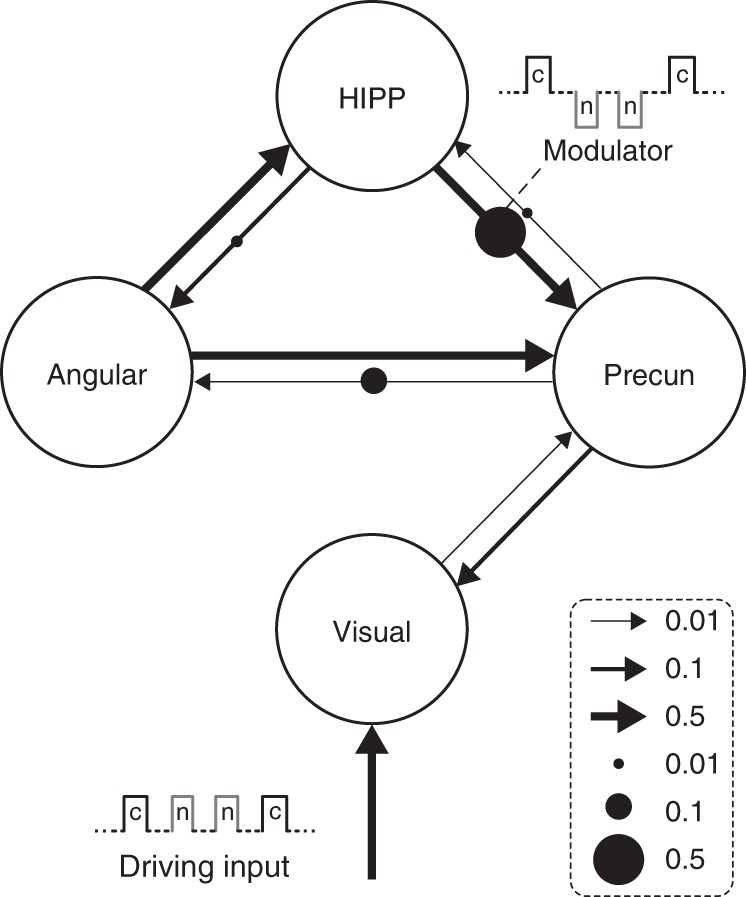
Winning model of DCM analyses considering modulations effect. This is the winning model of the space defined by adding modulators to the intrinsic connectivity model. Connectivity, driving input and modulator are coded in the thickness of line, and the size of arrow and circle, respectively, representing their effect size (c: continuing news clip, n: naïve news clip). HIPP = Hippocampus; Precun = Precuneus; Angular = Angular gyrus

Finally, the BMS procedure was performed on all the 64 competing models. As expected, the model which combines the effects of each winning family was selected (model 52), with exceedance probability of 0.29 (Fig. 3c). In this model, the strongest endogenous connections are the ones from angular gyrus to precuneus and hippocampus, and from hippocampus to precuneus, where the latter received the most robust modulation from the task conditions (Fig. 4, Supplementary Tables 1 and 2). This winning model highlights the functional relevance of the anterior hippocampus–precuneus connection to episodic memory retrieval.

### Prediction of subsequent recall task performance

**Fig. 5:**
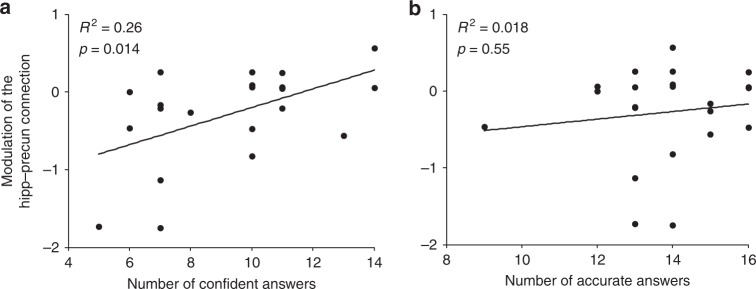
Correlation between DCM parameter and behaviour data. Correlation between the DCM parameter of the modulation of the effective connectivity from the hippocampus to the precuneus and (**a**) the number of confident answers, (**b**) the number of accurate answers derived from news recall task

We finally investigated the behavioural relevance of the network underlying involuntary memory retrieval. Following the news viewing tasks, participants responded to 18 questions related to the 9 continuing news clips (Supplementary Figure 1e). We investigated whether the DCM parameters predicted the number of confident answers and the number of accurate answers to these questions. We selected the modulation of the hippocampus to precuneus connection as it was modulated the most strongly during the news viewing task (Fig. 4). The corresponding parameter significantly correlated with the number of confident answers (*R*^2^ = 0.26, *p* *=* 0.014, Fig. 5a), but not the number of accurate answers (*R*^2^ = 0.018, *p* *=* 0.55, Fig. 5b). Of note, the number of confident answers and the number of accurate answers were correlated (*R*^2^ = 0.25, *p* = 0.017, Supplementary Figure 6). Because accurate answers may include correct guesses, we further sought to disambiguate confidence and accuracy. Confident answers that were incorrect—predominantly reflecting confidence but not accuracy—were still significantly correlated with the modulation parameters of hippocampus–precuneus connection (*R*^2^ = 0.26, *p* *=* 0.016, Supplementary Figure 7a). Confident and correct answers, presumably containing less guesses, did not show a significant correlation (*R*^2^ = 0.14, *p* = 0.087, Supplementary Figure 7b). These results thus suggest that a strengthened hippocampus to precuneus connectivity during memory retrieval predicts greater subsequent confidence in one’s memory, a core component of metacognition.

We further tested if this relationship was specific to the hippocampus-precuneus connection by regressing parameters of the other three modulations with behaviour data. None of these parameters were significantly correlated with either confidence or accuracy (*R*^2^ < 0.1, Supplementary Figure 8). Also of note, the correlation with behaviour was not present with functional connectivity between anterior hippocampus and precuneus (see section Functional connectivity analysis; Supplementary Figure 9, *R*^2^ < 0.1).

## Discussion

Our study provides several new insights into the neural mechanism of memory processes by positioning them in a naturalistic and dynamic context. We first show that passive viewing of the news clips that have personal, historical context robustly increases engagement of the anterior hippocampus, as well as a constellation of regions in the DMN, executive and control systems and cerebellum. Employing a systematic approach to construct model space based on DCM, we showed that the effects in the anterior hippocampus, precuneus and angular gyrus could be accounted for by increased, context-specific effective connectivity. Notably, the modulation of effective connectivity from the hippocampus to the precuneus during involuntary memory retrieval is a strong predictor of individuals’ confidence in their memories, but not the accuracy of their performance. Our findings thus provide a unique window into the dynamic interactions amongst this core network under an ecologically valid condition, and the associated metacognitive inference.

While voluntary memories can be strategically retrieved, involuntary memories arise spontaneously, informing present interactions with the world without conscious effort. In prior work, involuntary memory was often associated with emotional distress or unwanted memories^22,23^ and focused on its maladaptive manifestation in clinical disorders such as PTSD^3^. However, recent psychology research has revealed that involuntary memory is indeed a basic mode of remembering and occupies a large proportion of retrieval processes in daily life^24–26^. Functional neuroimaging studies have begun to probe the neural mechanism of involuntary memory, using static stimuli, such as images or images combined with sounds^21,27^. Such a design is generally consistent with the majority of episodic memory tasks, which predominately depend on arbitrary and static relationships between abstract stimuli, and often focus on a specific contextual feature of the stimuli^4–6^. However, memory processes engaged by static stimuli are arguably substantially different from naturally acquired involuntary memories, since the latter are more likely to involve complex, multimodal and dynamic information. The relative lack of focus on multimodality and dynamic scenes could introduce confounds. For example, failure to retrieve a particular unimodal feature does not necessarily equal with a failed retrieval, as the episodic memory could still be triggered by additional contextual features beyond the designated source^10^. As memory in every-day life involves rich contextual features and has an involuntary nature, there is increasing demand for an ecologically valid experimental paradigm.

There have been a number of functional imaging studies using naturalistic stimulus to investigate episodic memory retrieval^19,20^. While naturalistic stimuli offer an ecologically valid condition and richer contextual features for studying episodic memory, its unconstrained and dynamic nature poses challenges to identifying the neural correlates of specific cognitive processes. We here developed a experimental design to target a specific cognitive process, involuntary memory retrieval, embedded in a naturalistic context. The anterior hippocampus, precuneus, angular gyrus and superior medial frontal cortex were recruited. These brain networks underlying episodic memory retrieval have been widely reported in previous studies^9,10,20^. However, of note, the hippocampus is notoriously difficult to engage in traditional fMRI task designs using simple and abstract stimuli. Here, we observed robust hippocampal activations, which may reflect the rich audio–visual features offered by naturalistic stimuli. Our results further highlighted the functional differences along the anterior–posterior axis of the hippocampus—while animal studies and structural imaging analysis often focus on the hippocampal subfields based on its cytoarchitectonic appearance^7^, the anterior–posterior subdivision could be more relevant to human memory function and its impairments in neuropsychiatric disorders^28^.

Several recent studies employed a similar task design, with continuing vs. non-continuing viewing^29,30^. Some studies delivered naturalistic stimuli in a model-free manner without repetition^20,29^. Such unconstrained designs are challenging for the delineation of specific cognitive processes. Keidel and colleagues designed a similar hybrid blocked design using stand-up TV comedies^30^, which enabled them to test the specific effect of familiar narrative contexts on information encoding. We expect to see more studies in the future that embed the rich dynamic and contextual information offered by naturalistic stimuli in a rigorously controlled design.

The precuneus is a crucial player in the DMN. Previous studies have revealed that the involvement of DMN in a retrieval-related network arises from its interaction with hippocampus, supporting episodic memory retrieval processes^10,20^. However, some questions still remain elusive—namely, how does information propagate within this retrieval-related network? Here, we investigated the effective connectivity between anterior hippocampus, precuneus, angular gyrus and visual cortex. While most prior DCM studies defined the model space with a small number of models tightly constrained by one hypothesis, we here employed a broader model space^31,32^. The winning model possesses direct connections between the precuneus and the visual cortex, as well as between precuneus and all three other network nodes. The reciprocal connections from the angular gyrus and hippocampus to the precuneus are also significant: The precuneus hence serves as a network hub, receiving the sensory information from primary visual cortex and relaying this to the angular gyrus and hippocampus (Fig. 4). This hub role of the precuneus is also consistent more broadly in light of its wide-spread anatomical connectivity with cortical and subcortical structures^12,31,33,34^. While some have reported that minimal anatomical connections from precuneus to hippocampus^18^, we found strong effective connectivity between the anterior hippocampus and precuneus (Fig. 4). This robust effective connectivity might arise from indirect connections through other network nodes, rather than direct anatomical connections, revisiting prior notions that effective connectivity does not mandate direct anatomical connections^35^.

The employment of DCM allowed us to then study the modulation of prior context on these endogenous connections. The winning model shows that memory retrieval strongly modulates the hippocampus–precuneus connection, building on previous reports on the functional involvement of precuneus in memory retrieval, including allocating top-down attentional resources to retrieval processes^11^, familiarity-driven recognition^36^ and successful memory retrieval^12,14^. In addition, memory retrieval also weakly modulated the angular gyrus–precuneus and angular gyrus–hippocampus connections. These findings are consistent with other neuroimaging investigations of the functional relevance of angular gyrus in retrieval processes, including successful and subjective recollection^11,36^, bottom-up attention^11^, episodic memory buffer^37^, and its role as convergence zone, binding the different features of an episode into integrated representation^38^.

Metacognition of memory—the ability to monitor and evaluate memory—is an essential feature of human memory and at the core of higher-order cognition^39,40^. The accuracy of metacognitive beliefs crucially determines the outcomes of learning and decision-making^41^. While metacognitive performance is known to involve prefrontal cortex, recent neuroimaging and lesion studies have further revealed associations with the hippocampus and parietal structures, i.e., precuneus^15–17^. Our analyses showed that the modulator of the hippocampus–precuneus connection is strongly correlated with participants’ subsequent confidence of their memory (Fig. 5a, Supplementary Figure 7a); that is, the stronger the modulation of effective connectivity from the anterior hippocampus to the precuneus, the more confidence participants subsequently held in their memories, suggesting the contribution of this connection to metacognition of memory. On the other hand, modulations of the angular gyrus did not show a behavioural correlation (*R*^2^ < 0.1, Supplementary Figure 8), despite contributing to successful and subjective recollection in other paradigms^11,36^. Recollection confidence is likely a multifaceted process, involving subjective and voluntary appraisal, as well as automated and involuntary processes. It is possible that prefrontal mechanisms account for the subjective revaluation, the hippocampus–precuneus effective connectivity determines the ease and strength of the involuntary memory retrieval.

Note, this correlation with behaviour was not present when using standard functional connectivity analysis, i.e., statistical dependence between anterior hippocampus and precuneus BOLD signals (*R*^2^ < 0.1, Supplementary Figure 9). A lack of convergence is perhaps not surprising in light of the distinction between model-driven, dynamics measures of effective connectivity, as revealed by DCM, and static linear measures of correlation. The winning DCM model also explicitly incorporates the modulatory effect of memory retrieval, unique to the continued condition: This systems-level context-dependence is not captured by simple pair-wise statistical correlations and highlights the value of considering effective connectivity in network studies of brain function^42^.

Several caveats need to be kept in mind. First, the endogenous connectivity in our model space was informed by prior anatomical knowledge. As effective connectivity does not necessarily depend on direct anatomical connections, future work could further extend the model space to account for all possible endogenous connections. However, prior work has shown that effective connectivity is more likely when there exists a stronger corresponding anatomical link^43^. Second, our results are derived from a relatively modest sample and could benefit from validation in a larger cohort. Third, as the separation of involuntary and voluntary memory is considerably artificial, recent neuroscience studies reveal that both of these two modes operate on the same underlying episodic memory system, suggesting that they share the same basic encoding and maintenance factors and occasionally cooperate^1^. Fourth, the continuing condition rests upon the retrieval of recently encoded memory, as well as encoding new information present in the dynamic stimuli: To understand the second half of the continuing news clips, participants integrate these newly encoded percepts into the context retrieved from the first half. However, memory encoding is also engaged by viewing the naïve clips. Thus the contrast between the continuing and naïve conditions predominantly infers involuntary retrieval processes. Fifth, we did not randomise the new clips between the two conditions. The paradigm was designed to be applicable for future longitudinal, clinical studies, and used fixed sets of news clips across all subjects and conditions rather than a pseudorandomised design. A fixed design reduces inter-individual variability, and thus benefits individual diagnostic and reliability analyses. However, a fixed design might be biased by differences between clips. In addition, the motivation for clinical application also caps the duration of the paradigm and consequently the number of clips that could be included, in order to minimise fatigue and motion in clinical populations. Nonetheless, the news clips selected for two conditions did not differ in a comprehensive set of audio and video features (Supplementary Table 3). Permutations analyses further confirmed that the differences between the two conditions were not due to random difference between clips (Supplementary Figure 10). Finally, as DCM requires a priori hypothesis of a few key regions of interest^44^, our analyses thus focused on the selected brain regions that are usually implicated in the episodic memory retrieval. Recent developments in large-scale DCMs for resting-state fMRI data^45^, could permit future incorporate a greater number of regions in order to address complementary hypotheses about involuntary recall. Future work could extend these findings not only to inter-hemisphere communication and asymmetry of retrieval-related network during involuntary memory retrieval, but also to other dynamic stimuli, including dramatic film and spoken narratives, or even snippets of each participant’s own lived experience using embedded technologies^46^

Neuropsychiatric disorders often manifest with deficits in memory retrieval and meta-memory. Understanding the neuropathological correlates of memory deficits is thus of substantial clinical relevance. However, it is challenging to conduct functional neuroimaging study in clinical populations, if the paradigms involve training and high cognitive demands. Thus, our passive viewing paradigm is ideally suited for examining functional deficits in clinical conditions, as participants only need to freely view or listen to naturalistic stimuli^47,48^. The use of a computational method such as DCM method provides a way of investigating the underlying neural mechanism of these neurodegenerative disorders during natural viewing.

## Methods

### Participants

Participants comprised 23 volunteers (12 female, age 27.6 ± 5.0 years), with normal or corrected-to-normal vision who disavowed a neurological disorder or use of psychotropic medication. Data from one participant was excluded due to incorrect button press during the news recall task. The study received approval from the human ethics research committee of the QIMR Berghofer Medical Research Institute and was conducted according to National Health and Medical Research Council guidelines. Participants provided written informed consent and received an AUD $25 voucher for participation in this study.

### Stimuli and paradigm

News clips were purchased from Australian Broadcasting Corporation (ABC), on different topics including animal, local, politics, sports, science and business. Eighteen clips were selected for the neuroimaging experiments. The length of news clips ranged from 31 to 59 s (45.06 ± 6.67). Each clip was cut at an existing scene cut into two halves of similar length. Nine clips were selected for the continuing condition and nine for the naïve condition, matched for topic, valence, gender of newscaster and clip length. In addition, the two groups of clips do not differ in their audio and video features, including motion intensity, visual excitement, brightness and detailed emotion categorisations (uncorrected *p* > 0.05, two-tailed Student’s *t*-test; Supplementary Table 3, see Video/audio features of news clip part). The first halves of the 9 clips in the continuing condition were shown to the participants during the first viewing session (Supplementary Figure 1a). After a 10–15 min delay (see below), the second halves of all 18 news clips were shown to the participants (Supplementary Figure 1c). All news clips were interleaved by fixation periods of 10–12 s. The order of clips within each session (initial and continuing) was randomised for each participant.

The initial and subsequent news viewing sessions were interleaved with a face–name association task that is often used to study episodic memory^4,49^ (Supplementary Figure 1). This design serves for two purposes: (1) creating a delay and distraction between the first and second news viewing sessions and (2) allowing comparison of our naturalistic paradigm to a conventional task. Specifically, 66 faces with neutral expressions and popular first names were selected from materials used in previous studies^50^, which were taken from a public face database, Psychological Image Collection at Stirling (PICS; http://pics.psych.stir.ac.uk/2D_face_sets.htm). The face–name stimuli consisted of a face shown on a black background with a fictional first name printed underneath the face. Faces and names were randomly paired. The face–name association task has two sessions: face-learning and face-recall session. In the face-learning session, participants learned 30 face–name pairs randomly selected from the face–name database—each pair was shown three times in total (Supplementary Figure 1b). The order of face–name pairs was randomised, and the intervals between faces ranged from 3.5 to 3.75 s. In the face recall session, participants completed 60 face–name association tests, including 20 faces previously presented in the face-learning task and 10 novel faces (Supplementary Figure 1d). Each face was shown twice and each time with two similar names. Participants were requested to choose the name that they regarded as the correctly associated one. The order of presented faces was randomised, and intervals between trials ranged from 5 to 5.2 s. Note that the accuracy for each participant was calculated only for the learned faces.

We also sought to understand how brain network interactions during the film clip viewing predicted the confidence and accuracy of future recall. Therefore, after the two free-viewing sessions, participants completed a news recall task: They were asked to select the one correct choice out of the two given possibilities regarding the content of the news clips. For this task, participants were first shown a scene from a continuing news clip and then answered a question about this news by selecting between two alternative choices (Supplementary Figure 1e). After each question, participants rated their confidence in their answer with a yes/no choice. Two questions were asked for each news clip in the continuing condition while none was asked for the ones in the naïve condition. The order of the 18 questions was randomised for each participant. Participants were not asked about the naïve clips since they were only presented partially and consequently subject to incomplete encoding. The recall accuracy of the news recall task did not correlate with the one of the face–name association task, possibly due to the ceiling effect of the latter (Supplementary Figure 11, *p* = 0.34).

### Video/audio features of news clip

We extracted low-level audiovisual properties of the news clips, as well as higher-level features capturing the emotion and saliency. Several visual features were extracted using established methods in Computer Vision. Specifically, in professional videos, the salience of an event can be conveyed by shot changes, such that a director can increase the frequency of shot changes (increase the event density) to heighten arousal^51^. We thus measured the average shot duration of each news clip^52^. Visual excitement is based on the average number of pixel changes between consecutive frames^52^. Motion intensity for each frame was calculated by first estimating the motion vectors of all the sub-regions and then averaging the moving distances of all these sub-regions^53^. The motion intensity was then averaged across frames to represent the motion for each news clip. Sharpness (the degree of blur or sharpness) and brightness (the average luminosity across the pixels) were extracted by pliers toolbox (http://github.com/tyarkoni/pliers)^54^.

Key audio features of news clips were then extracted^55–57^, using the MIR toolbox^58^. Energy describes the intensity of audio signals and was estimated from the amplitude of the associated waveform. Spectrum entropy indicates whether the audio curve contains predominant peaks or not. If the curve is flat, the entropy will be large. Conversely, if the curve only shows one sharp peak, the entropy is minimal. Silence ratio shows the proportion of very low energy over the whole signals. Zero crossing rate, a key feature to investigate the presence of human speech, counts the number of times the signal crosses the *X*-axis. Three final sound indices included the mean flux (the variation in the frequency spectrum over time), spectral centroid (the magnitude spectrum’s centre of mass of the signal—an index of sound brightness, such that a dark sound has a low value), and fund frequency (the frequency of the best pitch, which is related to evoked valence)^59^.

We then extracted higher-level features of news clips. The emotion feature was determined for the human faces contained in each frame by using the facial emotion recognition API of Indico (https://indico.io) via the pliers toolbox (http://github.com/tyarkoni/pliers)^54^. The probability that the human faces in the frame express each of six emotions, including angry, sad, neutral, surprise, fear, and happy, was evaluated, respectively. For each emotion and news clip, the probabilities were then averaged across frames, resulting in six probabilities representing six emotions for each clip. Moreover, as higher saliency can attract the attention of audience and increase the arousal level accordingly, the feature saliency was also calculated by the pliers toolbox (http://github.com/tyarkoni/pliers)^54^. The saliency map of each frame was first calculated using the algorithm in pliers (http://github.com/tyarkoni/pliers), and then the maximum values for all the saliency maps were averaged across frames to represent the saliency level of each news clip.

### Image acquisition and preprocessing

All structural and functional images were acquired from a whole-body 3T Siemens Prisma MRI Scanner. A high-resolution T1-weighted MP2RAGE structural image was acquired for each participant with following parameters: TR = 4000 ms, TE = 2.96 ms, FA1 = 6°, FA2 = 7°, FOV = 240 mm × 256 mm, and voxel resolution 1 mm × 1 mm × 1 mm. The functional images were acquired with a multiband echo-planar imaging (EPI) sequence: TR = 1240 ms, TE = 32 ms, FA = 67°, FOV = 215 mm × 215 mm, multiband acceleration factor = 3, iPAT factor = 2, voxel resolution 2.5 mm × 2.5 mm × 2.5 mm and 60 slices. Functional images were preprocessed using Statistical Parametric Mapping toolbox (SPM12, Welcome Department of Imaging Neuroscience, Institute of Neurology, London). The preprocessing pipeline included slice timing correction and realignment using a six-parameter linear transformation, co-registration, normalisation, spatial smoothing with 4 mm full width half maximum Gaussian kernel, and band pass filtering (0.0085–0.15 Hz). After band pass filtering, nuisance covariates including WM, CSF and motion parameters were then regressed out using the Data Processing Assistant for Resting-state fMRI software (DPARSF) to reduce potential effects of physiological confounds^60^. Note that functional images without band pass filtering and nuisance regression were used for GLM and DCM analyses, but a high-pass filter of 128 s was built within the GLM/DCM analyses. Images preprocessed with these two steps were used for inter-subject correlation (ISC) analysis (see below).

### Voxel-wise ISC analysis

We first employed voxel-wise ISC analyses to identify brain regions showing consistent neural activity across participants during news viewing (either first or second news viewing scan), to benchmark our paradigm against prior research^61^ (Supplementary Figure 12 and Supplementary Note 2). The ISC analyses and statistical tests were performed using the ISCtoolbox^62^, and were applied separately to the functional images of each news clip in first and second news viewing. For each news clip, voxel-wise ISC maps were derived by calculating the Pearson correlation of time series between each pair of participants on a voxel-by-voxel basis.

We then assessed the significance of ISC results using an established non-parametric method implemented by ISCtoolbox^62^, as used by previous studies^47,63,64^. Specifically, as correlation maps derived from each pair of participants were not independent, we performed a non-parametric permutation test with 10,000,000 randomisations to identify statistically significant results. Note that the total number of potential unique permutations is *T*^*n*^ (where *T* = 15 is the number of volumes of shortest news clip and *n* = 22 is the number of subjects), resulting in 7.5 × 10^25^ possible unique combinations, suggesting that the null can be appropriately represented by this process. In each iteration, the BOLD time series of each participant was circularly shifted by a random number of volumes separately and ISC was recomputed on the shifted data. This preserves the temporal autocorrelation in the BOLD signals but destroys the cross correlations between participants. To determine voxels with significant ISC values, we first computed the *p*-values of each voxel based on the null distribution and then adjusted these *p*-values using a false discovery rate (FDR) to correct for multiple comparisons, with a FDR-corrected threshold of *p* < 0.01. To define the group average ISC map for first viewing, continuing and naïve conditions during second viewing separately, we simply averaged the thresholded ISC maps (FDR-corrected *p* < 0.01) for all the news clips belonging to each condition.

### GLM analysis of functional images

Functional MRI images were analysed using SPM12. For the news viewing scans, GLM was first applied to each participant in a first level GLM analysis. In the first news viewing session, there was one condition—viewing the first half of news clips; in the second news viewing session, there were two conditions—continuing and naïve news conditions. In the news viewing task, each news clip was assigned as a regressor in the design matrix. The six head motion parameters derived from the realignment stage were included as nuisance covariates.

Group-level activations were identified by one-sample *t*-tests with random effects analysis using a second level GLM. For the first viewing, we defined one contrast of interest to examine brain activation in relation to news viewing. For the second viewing session, we defined four contrasts of interests: ‘continuing news clips’, ‘naïve news clips’, ‘continuing-naïve’ and ‘naïve-continuing’. Significant brain activations were defined using a family-wise correction (FWE) corrected with an initial search threshold of *p* < 0.001 for voxel height and a final cluster-wise FWE-corrected threshold of *p* < 0.001. To further validate that the different activations between continuing and naïve conditions are not due to chance differences between the news clips in these two conditions, a permutation test was performed for GLM analysis, where in each iteration for each subject we randomly selected five news clips from each condition, respectively, and repeated the procedure described above. This permutation test was conducted for the continuing vs. naïve contrast and included 1000 iterations (Supplementary Figure 10).

In addition, we also replicated the GLM results by assigning the two conditions as two regressors and modelled the contrast between the two (Supplementary Figure 13).

Furthermore, for the first-level GLM analysis of face recall task, there were three conditions—novel faces, learned and correct faces, learned and incorrect faces, resulting in three task-related regressors in the design matrix (novel, correct and incorrect faces). The six head motion parameters derived from the realignment stage were also included as nuisance covariates. For the second level GLM analysis, we defined four contrasts: the ‘correct faces’, ‘novel faces’, ‘correct-novel’ and ‘novel-correct’. Significant brain activations were defined using a cluster-wise FWE of *p* < 0.001 for cluster following a *p* < 0.001 height threshold. To compare the level of functional activations in retrieval-related contrast between the face recall and news viewing paradigms, we extracted the average *z*-scores of all voxels within the six brain regions that were commonly engaged during episodic memory retrieval, namely, the precuneus and bilateral hippocampus, angular gyrus and medial prefrontal cortex. To avoid potential bias, we applied the same masks (based on the 90 fROIs atlas and the AAL atlas) to both results.

### DCM analysis during second news viewing

DCM provides a computational framework to investigate effective connectivity between cortical regions by combining dynamic models of underlying neuronal states with a hemodynamic forward model^35^. Here, DCM was applied to examine effective connectivity between the regions of interests (ROIs) identified in the GLM analysis of the second news viewing. DCM is most efficient and informative when applied to a relatively small number of regions that are of specific interest to the cognitive domain in question. We thus focused on regions that have been consistently reported in the literature of episodic memory, including anterior hippocampus, precuneus, angular gyrus and secondary visual cortex and that additionally showed strong experimental effects in our data (see Results). Following standard criteria^43^ and consistent with previous DCM studies^32,65^, we defined the centre of each ROI as the peak of functional activation from the group-level GLM results of the ‘continuing > naïve’ contrast, verified by the Automated Anatomical Labelling Atlas (AAL)^66^. Starting from the peak of group-level activation, the nearest subject-specific peak was identified within the 4 mm radius of the group-level peak for each subject. Next, the subject-specific ROIs corresponded to all significant voxels that survived a threshold of *p* < 0.05 (uncorrected) within the 4 mm radius sphere centred over each subject-specific peak. The time series of these ROIs were then extracted for each subject. We here mainly focused on the effective connectivity between ROIs in the left hemisphere, since functional activations were stronger on the left than the right. As a further step, we also performed the same DCM analysis between ROIs in the right hemisphere (Supplementary Figure 5). Inter-hemisphere communication and asymmetry were beyond the scope of this study.

DCM is a hypothesis-driven framework that operates on a user-defined model space, specified through the choice of driving input, intrinsic effective connectivity and contextual modulations. The driving input consisted of all the 18 news clips (either continuing or naïve news clips), while the prior viewing of the first half of the clip (contrast of continuing > naïve viewing) served as a contextual modulator. We constrained the driving input of news clips to enter the network via a visual ROI. Although the news clips also have auditory information, the time courses of the visual and auditory ROIs are highly correlated (0.74 ± 0.087). For model simplicity, we thus present the DCM analysis with driving input to the visual ROI only.

Accounting for all possible combinations of intrinsic connections and modulations in one large model space would yield large model space, causing a substantial computational burden and a likely flat model likelihood. Our DCM analysis hence proceeded in two steps following the methods recommended by Di and Biswal^31^, Goulden et al.^32^, and Breakspear et al. ^67^, as implemented in SPM12. The first step only concerned the intrinsic effective connectivity between ROIs, thus the different models were defined by varying the endogenous connectivity parameters (DCM A-matrix). Prior knowledge about anatomical connections can provide important structural constraints to refine model space^44^. Thus, based on the anatomical connections derived from macaque monkey and human studies, we constructed the model space by considering four sets of connectivity families, including connections between (1) visual cortex and precuneus, (2) precuneus and hippocampus, (3) angular gyrus and precuneus and (4) angular gyrus and hippocampus (Fig. 2a)^8,12,13,18^. Anatomical studies found that occipital visual areas show strong structural connections with the precuneus^12,18^, but not substantially with either the angular gyrus nor hippocampus^13^. Thus, for the (1) visual connection family, we only specified connections between visual cortex and precuneus in our model space (Fig. 2a, right panel). We also ensured that models with a connection from precuneus to visual also had a backward connection, as visual cortex is the input region. Moreover, while it has been argued that hippocampus has weak or no direct structural connections with the precuneus^12,18^, a large number of functional imaging studies have revealed strong functional connectivity between these two regions^8,10,12^. Based on the strong functional connectivity between hippocampus and precuneus, we thus specified the (2) precuneus–hippocampus connection family in our model space (Fig. 2a, right panel). Finally, since there are strong structural connections between angular gyrus and precuneus, or angular gyrus and hippocampus^12,13^, there was no reason to construct a model space without (3) angular gyrus–precuneus, or (4) angular gyrus–hippocampus connection families (Fig. 2a, right panel). Combinations of these four types of families resulted in 2 × 4 × 3 × 3 = 72 models. Bayesian model selection (BMS) with random effects analysis was employed to select the winning model after balancing the fit of data and the model complexity^68^. BMS was first performed on the four types of families^69^, and then on all 72 models^31^.

Based on the winning model of endogenous connectivity from this first analysis (see above), we then accounted for the modulatory effects evoked by the continuing > naïve condition (DCM B-matrix). As episodic retrieval has been shown to engage hippocampus, precuneus and angular gyrus^10^, we considered that any endogenous connection between these three regions (forward and backward) could be modulated, resulting in three sets of modulator families (Fig. 3a). Combinations of these three types of families resulted in 4 × 4 × 4 = 64 models. Again, we first performed the BMS on the three types of families^69^, and then on all the 64 models^31^. Performing family-wise BMS allows us to focus on specific characteristics of interest within each family while minimising the uncertainty about model characteristics defined by other parameters^69^. Combining family-wise BMS with a full model space comparison can allow one to understand the principle, individual features that best explain the data, whilst also seeing how these are combined into a single unique model.

### Correlation between DCM parameter and recall task performance

To identify whether the DCM parameters predicted the performance of the news recall task, we regressed the modulation parameters of the winning model with the recall behaviour data across participants. We considered two types of data that reflect how well participants remembered the contents of the news clips—the number of confident answers and the number of accurate answers.

### Functional connectivity analysis

We examined whether the functional connectivity between the anterior hippocampus and precuneus could predict the performance of the news recall tasks as the DCM effective connectivity. Specifically, the time series of these two ROIs (same as in the DCM analysis) were extracted from the preprocessed fMRI images (with band pass filtering and nuisance covariates regression) for each subject. Functional connectivity measures were then calculated as the Pearson’s correlation between the two time series for each participant and then regressed against the recall behaviour performances across participants.

### Hippocampus parcellation

Resting state fMRI data of same subjects were adopted to generate the functional parcels for hippocampus via Craddock algorithm^70^. The left hippocampal mask was derived from AAL atlas and used for confining the parcellation results to left hippocampus. The cluster number was set to 4 (Supplementary Figure 14). In addition, we chose the combination of two-level clustering approach with temporal similarity metric, which outperforms other functional parcellation strategies in previous study^70^.

### Code availability

The custom code used to generate the results in this study is available from the corresponding author upon reasonable request.

## Electronic supplementary material

Supplementary Information

Peer Review File

## Data Availability

The data supporting the findings of this study are available from the corresponding author upon reasonable request.

## References

[1] Berntsen D (2010). The unbidden past: Involuntary autobiographical memories as a basic mode of remembering. Curr. Dir. Psychol. Sci..

[2] Rasmussen AS, Berntsen D (2011). The unpredictable past: spontaneous autobiographical memories outnumber autobiographical memories retrieved strategically. Conscious Cogn..

[3] Rubin DC, Boals A, Berntsen D (2008). Memory in posttraumatic stress disorder: properties of voluntary and involuntary, traumatic and nontraumatic autobiographical memories in people with and without posttraumatic stress disorder symptoms. J. Exp. Psychol..

[4] Kirwan CB, Stark CE (2004). Medial temporal lobe activation during encoding and retrieval of novel face‐name pairs. Hippocampus.

[5] Vilberg KL, Moosavi RF, Rugg MD (2006). The relationship between electrophysiological correlates of recollection and amount of information retrieved. Brain Res..

[6] Zeithamova D, Dominick AL, Preston AR (2012). Hippocampal and ventral medial prefrontal activation during retrieval-mediated learning supports novel inference. Neuron.

[7] Duvernoy H. M. *The Human Hippocampus: Functional Anatomy, Vascularization and Serial Sections with MRI* (Springer, New York, 1998).

[8] Vincent JL (2006). Coherent spontaneous activity identifies a hippocampal-parietal memory network. J. Neurophysiol..

[9] Kim H (2010). Dissociating the roles of the default-mode, dorsal, and ventral networks in episodic memory retrieval. Neuroimage.

[10] Rugg MD, Vilberg KL (2013). Brain networks underlying episodic memory retrieval. Curr. Opin. Neurobiol..

[11] Ciaramelli E, Grady CL, Moscovitch M (2008). Top-down and bottom-up attention to memory: a hypothesis (AtoM) on the role of the posterior parietal cortex in memory retrieval. Neuropsychologia.

[12] Cavanna AE, Trimble MR (2006). The precuneus: a review of its functional anatomy and behavioural correlates. Brain.

[13] Seghier ML (2013). The angular gyrus: multiple functions and multiple subdivisions. Neuroscientist.

[14] Wagner AD, Shannon BJ, Kahn I, Buckner RL (2005). Parietal lobe contributions to episodic memory retrieval. Trends Cogn. Sci..

[15] Baird B, Smallwood J, Gorgolewski KJ, Margulies DS (2013). Medial and lateral networks in anterior prefrontal cortex support metacognitive ability for memory and perception. J. Neurosci..

[16] McCurdy LY (2013). Anatomical coupling between distinct metacognitive systems for memory and visual perception. J. Neurosci..

[17] Allen M (2017). Metacognitive ability correlates with hippocampal and prefrontal microstructure. Neuroimage.

[18] Buckner RL, Andrews‐Hanna JR, Schacter DL (2008). The brain’s default network. Ann. N. Y. Acad. Sci..

[19] Chadwick MJ, Hassabis D, Weiskopf N, Maguire EA (2010). Decoding individual episodic memory traces in the human hippocampus. Curr. Biol..

[20] Chen J (2016). Accessing real-life episodic information from minutes versus hours earlier modulates hippocampal and high-order cortical dynamics. Cereb. Cortex.

[21] Hall NM, Gjedde A, Kupers R (2008). Neural mechanisms of voluntary and involuntary recall: a PET study. Behav. Brain Res..

[22] Clark I, Holmes E, Woolrich M, Mackay C (2016). Intrusive memories to traumatic footage: the neural basis of their encoding and involuntary recall. Psychol. Med..

[23] Gvozdanovic GA, Stämpfli P, Seifritz E, Rasch B (2017). Neural correlates of experimental trauma memory retrieval. Hum. Brain Mapp..

[24] Berntsen D. *Involuntary Autobiographical Memories: An Introduction to the Unbidden Past* (Cambridge University Press, Cambridge, England, 2009).

[25] Mace J (2007). New Perspectives in Cognitive Psychology. Involuntary Memory.

[26] Rubin DC, Berntsen D (2009). The frequency of voluntary and involuntary autobiographical memories across the life span. Mem. Cogn..

[27] Hall SA (2014). The neural basis of involuntary episodic memories. J. Cogn. Neurosci..

[28] Poppenk J, Evensmoen HR, Moscovitch M, Nadel L (2013). Long-axis specialization of the human hippocampus. Trends Cogn. Sci..

[29] van Kesteren MT, Fernández G, Norris DG, Hermans EJ (2010). Persistent schema-dependent hippocampal-neocortical connectivity during memory encoding and postencoding rest in humans. Proc. Natl Acad. Sci..

[30] Keidel JL, Oedekoven CS, Tut AC, Bird CM (2017). Multiscale integration of contextual information during a naturalistic task. Cereb. Cortex.

[31] Di X, Biswal BB (2014). Identifying the default mode network structure using dynamic causal modeling on resting-state functional magnetic resonance imaging. Neuroimage.

[32] Goulden N (2012). Reversed frontotemporal connectivity during emotional face processing in remitted depression. Biol. Psychiatry.

[33] Hagmann P (2008). Mapping the structural core of human cerebral cortex. PLoS Biol..

[34] Fransson P, Marrelec G (2008). The precuneus/posterior cingulate cortex plays a pivotal role in the default mode network: evidence from a partial correlation network analysis. Neuroimage.

[35] Friston KJ, Harrison L, Penny W (2003). Dynamic causal modelling. Neuroimage.

[36] Vilberg KL, Rugg MD (2008). Memory retrieval and the parietal cortex: a review of evidence from a dual-process perspective. Neuropsychologia.

[37] Baddeley A (2000). The episodic buffer: a new component of working memory?. Trends Cogn. Sci..

[38] Shimamura AP (2011). Episodic retrieval and the cortical binding of relational activity. Cogn. Affect. Behav. Neurosci..

[39] Fleming SM, Lau HC (2014). How to measure metacognition. Front. Hum. Neurosci..

[40] Fernandez-Duque D, Baird JA, Posner MI (2000). Executive attention and metacognitive regulation. Conscious Cogn..

[41] Flavell JH (1979). Metacognition and cognitive monitoring: a new area of cognitive–developmental inquiry. Am. Psychol..

[42] Friston KJ (2011). Functional and effective connectivity: a review. Brain Connect..

[43] Stephan KE, Tittgemeyer M, Knösche TR, Moran RJ, Friston KJ (2009). Tractography-based priors for dynamic causal models. Neuroimage.

[44] Stephan KE (2010). Ten simple rules for dynamic causal modeling. Neuroimage.

[45] Razi A (2017). Large-scale DCMs for resting-state fMRI. Network Neurosci..

[46] Nielson DM, Smith TA, Sreekumar V, Dennis S, Sederberg PB (2015). Human hippocampus represents space and time during retrieval of real-world memories. Proc. Natl Acad. Sci..

[47] Guo CC, Nguyen VT, Hyett MP, Parker GB, Breakspear MJ (2015). Out-of-sync: disrupted neural activity in emotional circuitry during film viewing in melancholic depression. Sci. Rep..

[48] Ren Y (2016). Assessing the effects of cocaine dependence and pathological gambling using group-wise sparse representation of natural stimulus FMRI data. Brain Imaging Behav..

[49] Sperling RA (2001). Encoding novel face‐name associations: a functional MRI study. Hum. Brain Mapp..

[50] Di Giorgio E, Leo I, Pascalis O, Simion F (2012). Is the face-perception system human-specific at birth?. Dev. Psychol..

[51] Zettl H. *Sight, Sound, Motion: Applied Media Aesthetics* (Wadsworth, Belmont, 2013).

[52] Wang HL, Cheong LF (2006). Affective understanding in film. IEEE Trans. Circuits Syst. Video Technol..

[53] Barjatya A (2004). Block matching algorithms for motion estimation. IEEE Trans. Evol. Comput..

[54] McNamara Q., De La Vega, A. & Yarkoni, T. (eds). Developing a comprehensive framework for multimodal feature extraction. *Proc. 23rd ACM SIGKDD International Conference on Knowledge Discovery and Data Mining* (ACM, New York, 2017).

[55] Canini L, Benini S, Leonardi R (2013). Affective recommendation of movies based on selected connotative features. IEEE Trans. Circuits Syst. Video Technol..

[56] Tzanetakis G, Cook P (2002). Musical genre classification of audio signals. IEEE Trans. Speech Audio Process..

[57] Koelstra S (2012). Deap: a database for emotion analysis; using physiological signals. IEEE Trans. Affect. Comput..

[58] Lartillot, O. & Toiviainen, P. (eds). A Matlab toolbox for musical feature extraction from audio. International Conference on Digital Audio Effects, Bordeaux (AudioLab, University of York, York, 2007).

[59] Picard R. W. *Affective Computing* (MIT Press, Cambridge, MA, USA, 1995).

[60] Chao-Gan Y, Yu-Feng Z (2010). DPARSF: a MATLAB toolbox for “pipeline” data analysis of resting-state fMRI. Front. Syst. Neurosci..

[61] Hasson U, Nir Y, Levy I, Fuhrmann G, Malach R (2004). Intersubject synchronization of cortical activity during natural vision. Science.

[62] Kauppi, J.-P., Pajula, J. & Tohka, J. A versatile software package for inter-subject correlation based analyses of fMRI. *Front. Neuroinform.* **8**, 2 (2014). 10.3389/fninf.2014.00002 PMC3907702 24550818

[63] Nguyen VT (2016). Distinct cerebellar contributions to cognitive-perceptual dynamics during natural viewing. Cereb. Cortex.

[64] Pajula J, Tohka J (2014). Effects of spatial smoothing on inter-subject correlation based analysis of FMRI. Magn. Reson. Imaging.

[65] Nguyen VT, Breakspear M, Hu X, Guo CC (2016). The integration of the internal and external milieu in the insula during dynamic emotional experiences. Neuroimage.

[66] Tzourio-Mazoyer N (2002). Automated anatomical labeling of activations in SPM using a macroscopic anatomical parcellation of the MNI MRI single-subject brain. Neuroimage.

[67] Breakspear M (2015). Network dysfunction of emotional and cognitive processes in those at genetic risk of bipolar disorder. Brain.

[68] Stephan KE, Penny WD, Daunizeau J, Moran RJ, Friston KJ (2009). Bayesian model selection for group studies. Neuroimage.

[69] Penny WD (2010). Comparing families of dynamic causal models. PLoS Comput. Biol..

[70] Craddock RC, James GA, Holtzheimer PE, Hu XP, Mayberg HS (2012). A whole brain fMRI atlas generated via spatially constrained spectral clustering. Hum. Brain Mapp..

