# Supplementary material for: Effective connectivity of the anterior hippocampus predicts recollection confidence during natural memory retrieval
Source: Nat Commun. 2018 Nov 19;9:4875. doi: 10.1038/s41467-018-07325-4 (PMC6242820; doi:10.1038/s41467-018-07325-4)
Supplement: Supplementary file 1 — Supplementary Information [file 41467_2018_7325_MOESM1_ESM.pdf]

Supplementary Information

Effective connectivity of the anterior hippocampus predicts recollection confidence during natural memory retrieval

Ren et al.

Supplementary Note 1

GLM results of face recall task. GLM analyses were performed to identify regions related to face-name association task (Supplementary Figure 1d). Contrasts for both novel and correct face conditions revealed activations within primary and association visual cortices, fusiform gyrus, dorsolateral prefrontal cortex, supplementary motor and premotor cortex, visuo-motor coordination area, supramarginal gyrus and dorsal anterior cingulate cortex (Supplementary Figure 2a-b, $p<0.001$ for voxel height and FWE corrected $p<0.001$ for cluster extent). In the bidirectional contrast to assess difference between novel and correct faces, correct face-name pairs evoked stronger activations in the middle and posterior hippocampus, precuneus, orbital and superior medial frontal cortex, anterior and posterior cingulated cortex, superior and middle temporal gyrus, supramarginal gyrus, secondary visual cortex and somatosensory association cortex than novel face-name pairs (Supplementary Figure 2c, $p<0.001$ for voxel height and FWE corrected $p<0.001$ for cluster extent). Note that this paradigm did not activate the complete core network underlying episodic memory retrieval, namely, angular gyrus (1). Overall, functional activations by the face-name association were weaker than the news viewing paradigm.

While the face-name association task evoked activations in the middle and posterior hippocampus, our news viewing paradigm evoked stronger reponses in the anterior hippocampus. This differential involvement of hippocampal subregions might be explained by a posterior-anterior hierarchy of information processing in the hippocampus. The anterior hippocampus was found to be engaged by scenes regardless of whether they are generated internally or externally, and forms internal representations of spatially coherent scenes, while posterior hippocampus appears to be particularly responsive to the perception of external visual cues (2).

Supplementary Note 2

Voxel-wise inter-subject correlation during news viewing. Free viewing of news clips evoked robust and consistent neural activity cross participants, as quantified by voxel-wise ISC analyses (Supplementary Figure 12a). Robust ISC was present in primary and association visual cortices, primary and association auditory cortices, fusiform gyrus, precuneus and premotor cortex during all three news viewing conditions. ISC was slightly stronger during the continuing and first than the naïve viewing, across several brain regions including the hippocampus (Supplementary Figure 12b), although these effects did not reach statistical significance.

Supplementary Figures and Tables

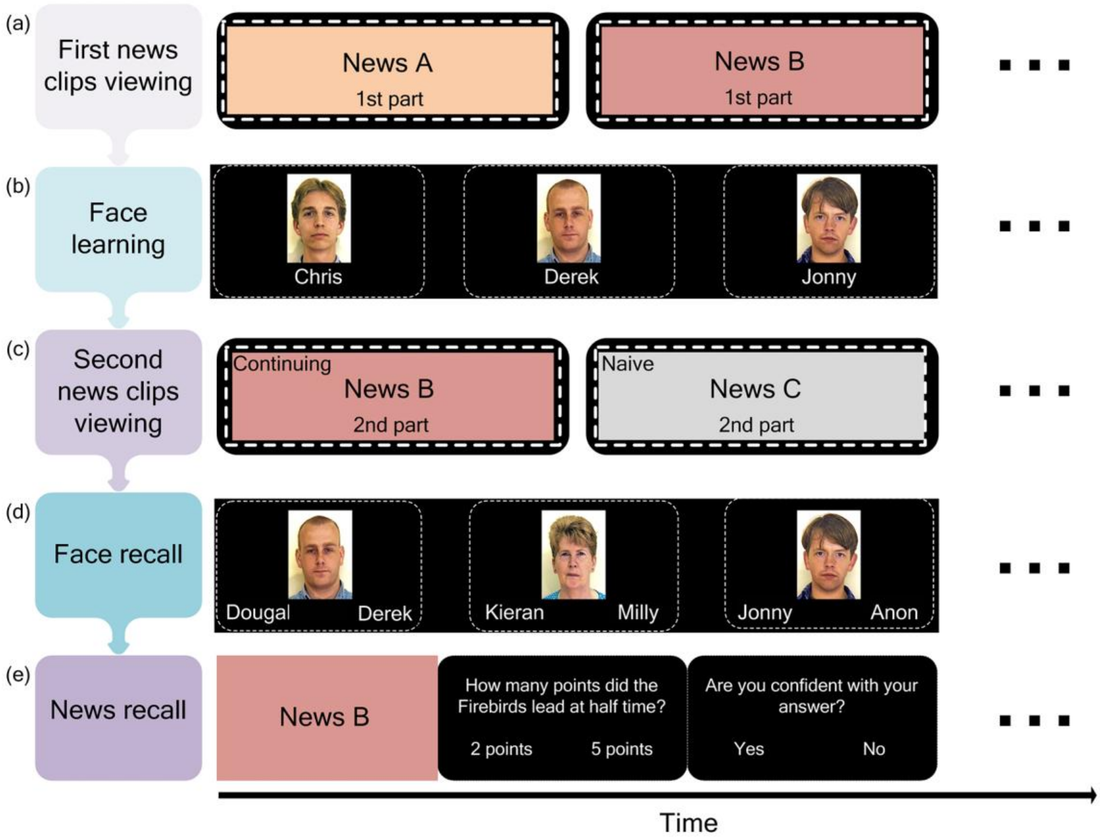

Supplementary Figure 1. Experiment design. (a) First news clips viewing session. (b) Face learning session. (c) Second news clips viewing session, including continuing and naïve conditions. (d) Face recall session. (e) News recall

session (*e.g.,* Q1. How many points did the Firebirds lead at the half time? 2 points or 5 points). The face images are from the public face database, Psychological Image Collection at Stirling (PICS; http://pics.psych.stir.ac.uk/2D_face_sets.htm), for illustrative purposes.

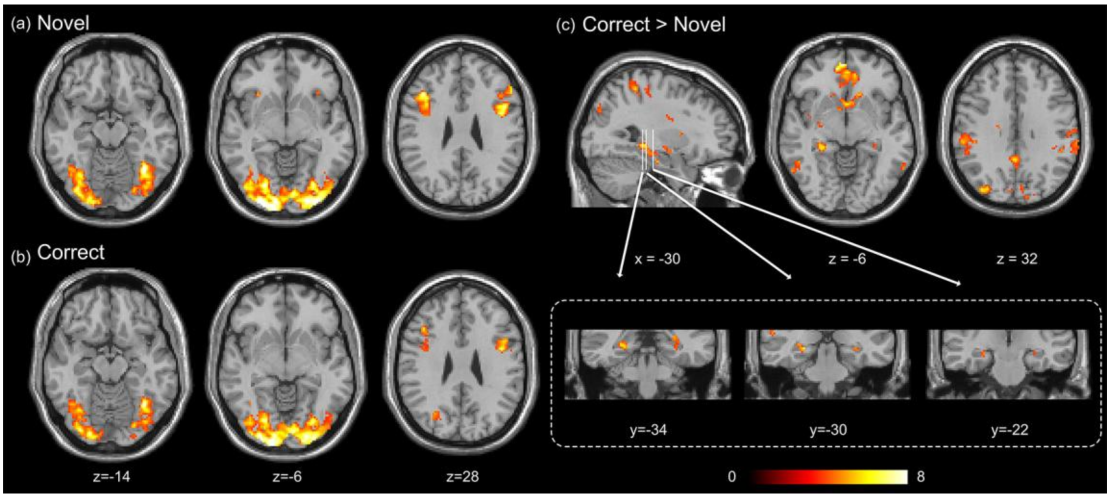

Supplementary Figure 2. General linear model (GLM) results of the face-name association recall task. Significantly activated brain regions under (a) novel, (b) learned and correct face-name conditions. (c) Brain regions that were more active to the correct faces than novel faces. Significant voxels were identified using a FWE cluster-corrected threshold $p<0.001$, with a voxel height defined with $p<0.001$.

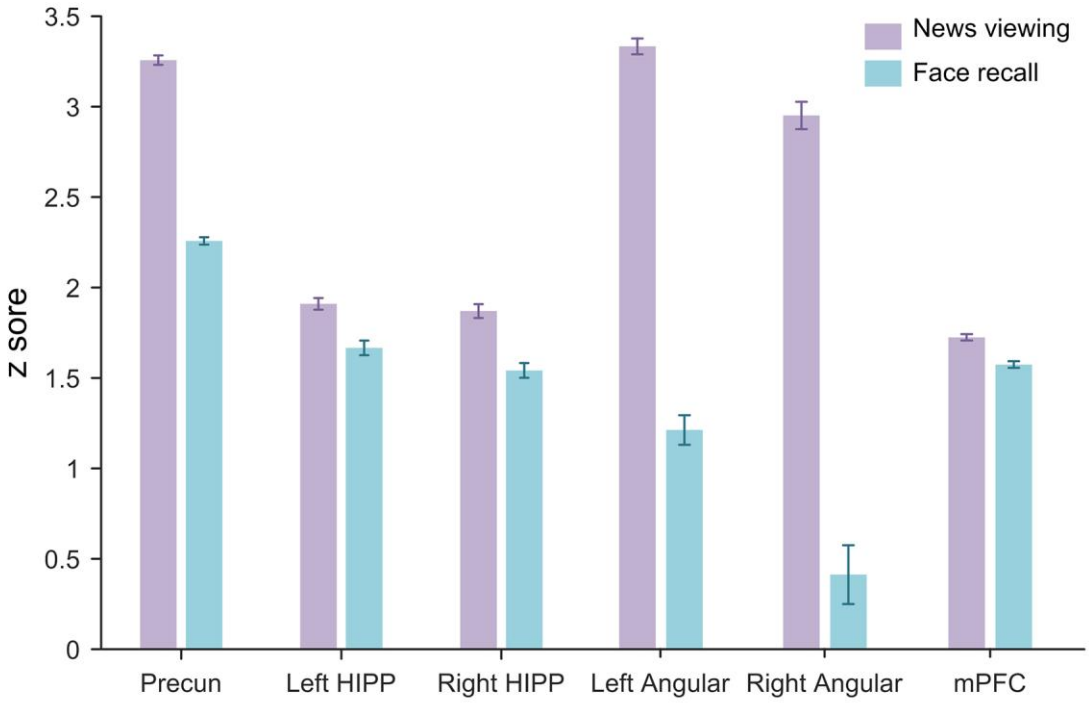

Supplementary Figure 3. The average z scores of brain regions that are consistently engaged in the retrieval-related contrast , for both news viewing and face recall paradigms (Precun: precuneus, HIPP: hippocampus, Angular: angular gyrus, mPFC: medial prefrontal cortex). Error bars signify SEM.

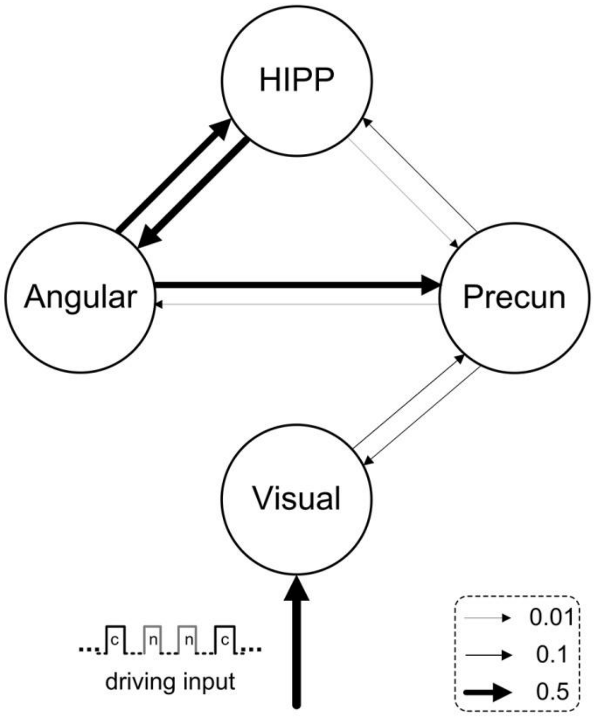

Supplementary Figure 4. Winning model defined by endogenous connectivity. Connectivity and driving input are coded in the thickness of line and the size of arrow, representing their effect size (c: continuing news clip, n: naïve news clip).

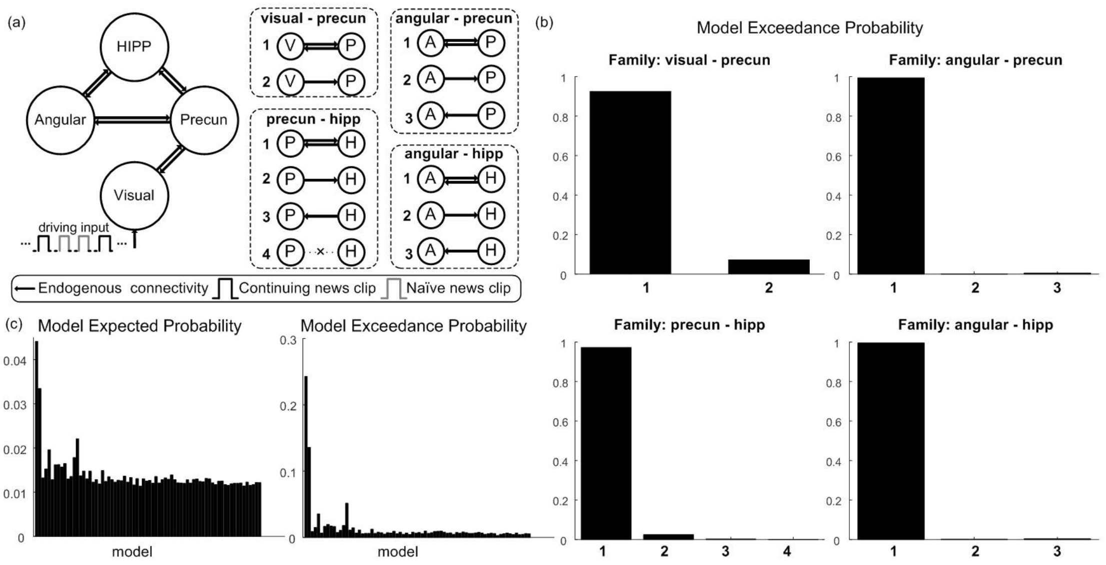

Supplementary Figure 5. DCM analyses of endogenous connectivity for second news clips viewing from ROI's in the right hemisphere. (a) Identifications of the model space in terms of four sets of connectivity families, including connections between R. visual and precuneus, R. angular gyrus and precuneus, R. precuneus and hippocampus, R. angular gyrus and hippocampus, resulting in 72 competing models. For all the models, the visual ROI receives the driving input. (b) Exceedance probability for the DCM model families in the BMS, including visual-precuneus families, angular gyrus-precuneus families, precuneus-hippocampus families, and angular gyrus-hippocampus families. The label of horizontal axis for each box corresponds to the label in (a). Bidirectional families are given first in each box. (c) Results of the BMS for all the 72 models. HIPP= R. Hippocampus; Precun= R. Precuneus; Angular= R. Angular gyrus.

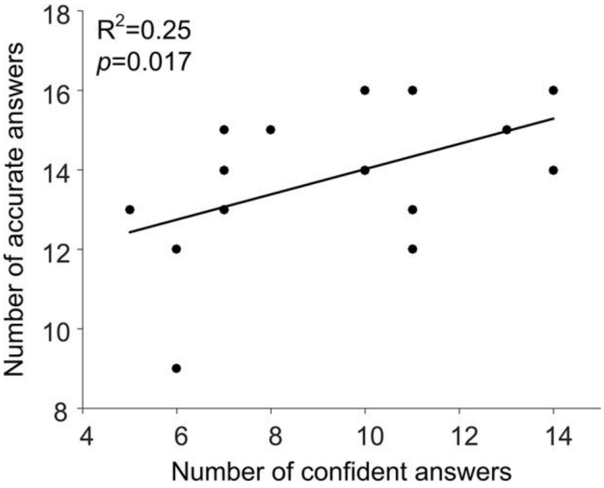

Supplementary Figure 6. Correlation between the number of confident answers and the number of accurate answers derived from news recall task.

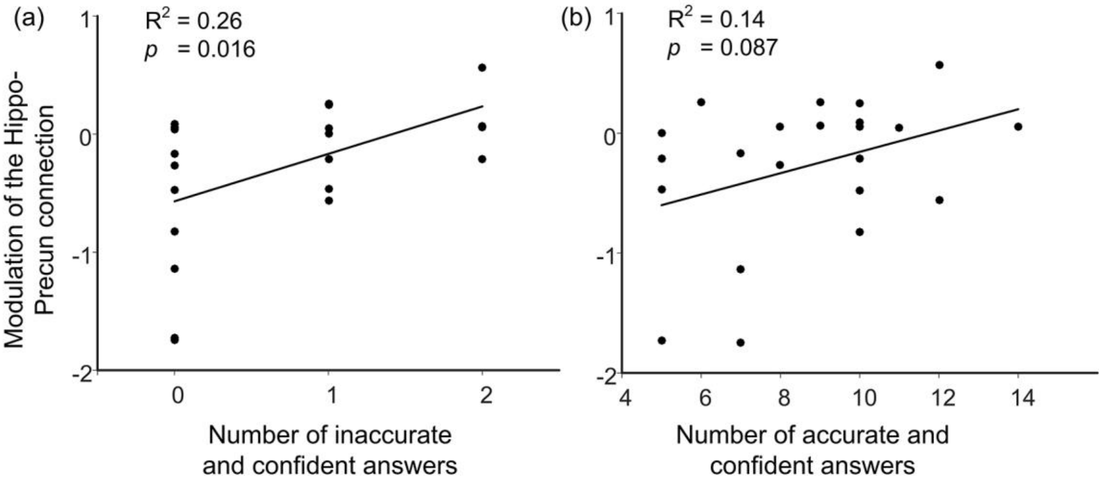

Supplementary Figure 7. Correlation between the DCM parameter corresponding to modulation of the effective connectivity from the hippocampus

to the precuneus and (a) the number of inaccurate and confident answers, (b) the number of accurate and confident answers derived from news recall task.

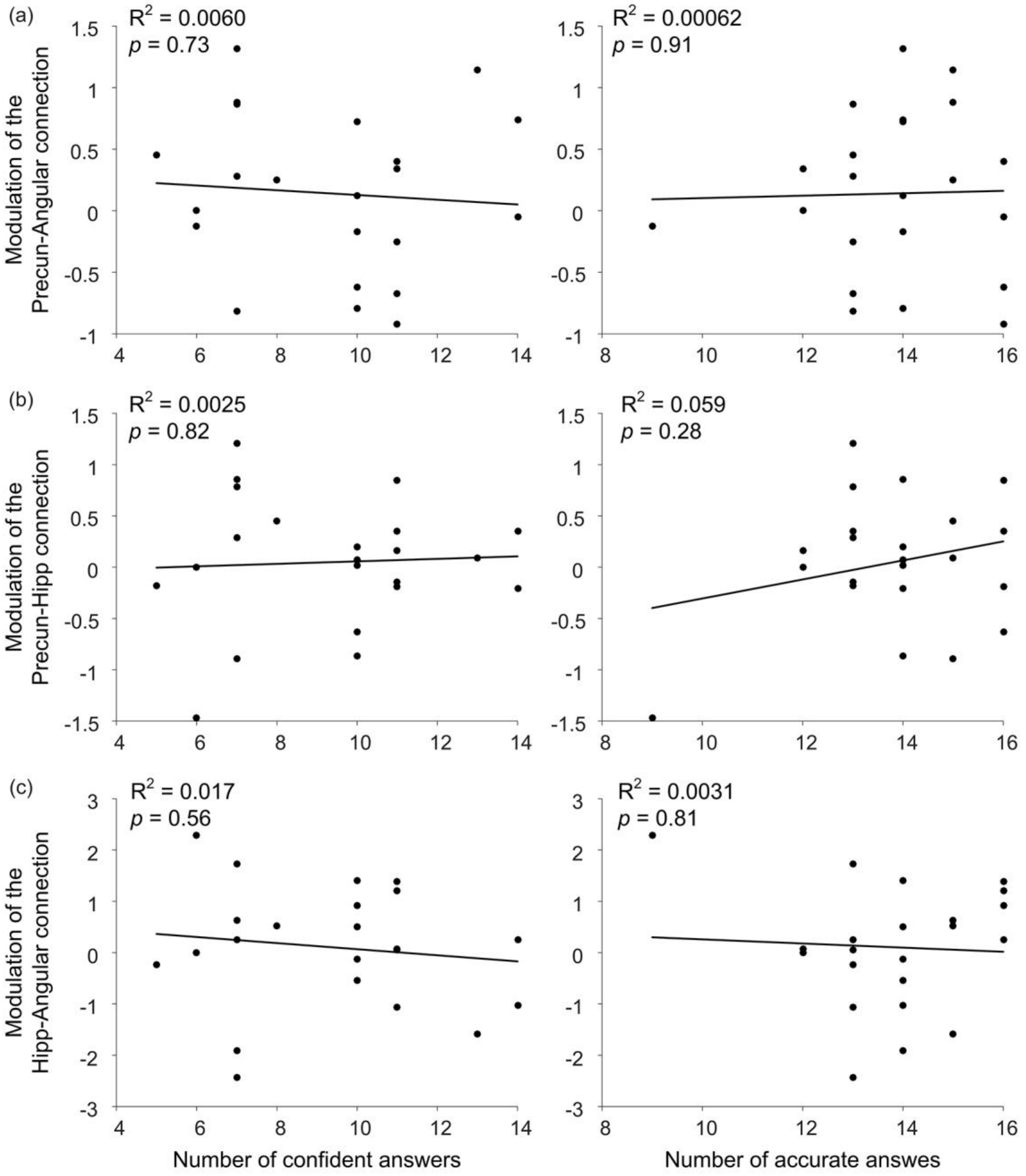

Supplementary Figure 8. Regressions of the DCM parameters corresponding to modulation of the effective connectivity (a) from the precuneus to the angular gyrus, (b) from the precuneus to the hippocampus, (c) from the hippocampus to angular gyrus against the number of confident answers (left column), or the number of accurate answers (right column) derived from the news recall task.

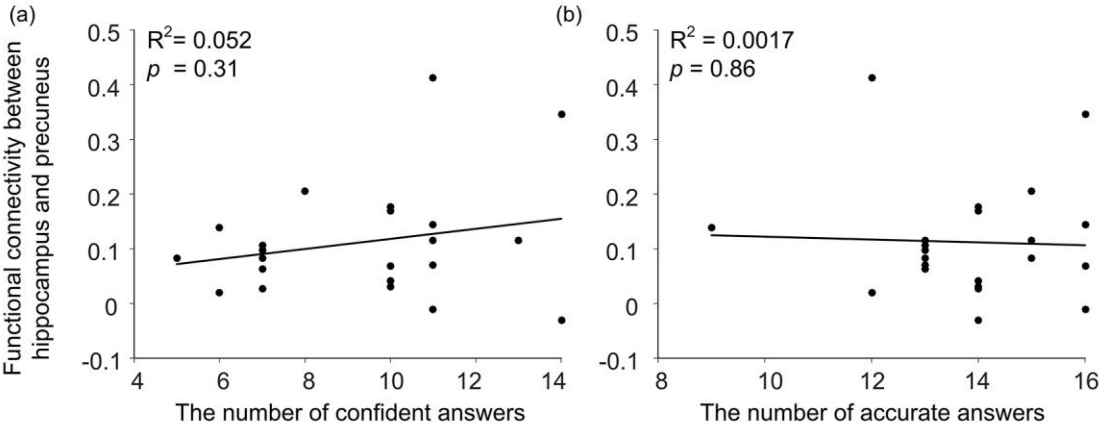

Supplementary Figure 9. Correlation between the hippocampus-precuneus functional connectivity and (a) the number of confident answers, (b) the number of accurate answers derived from news recall task.

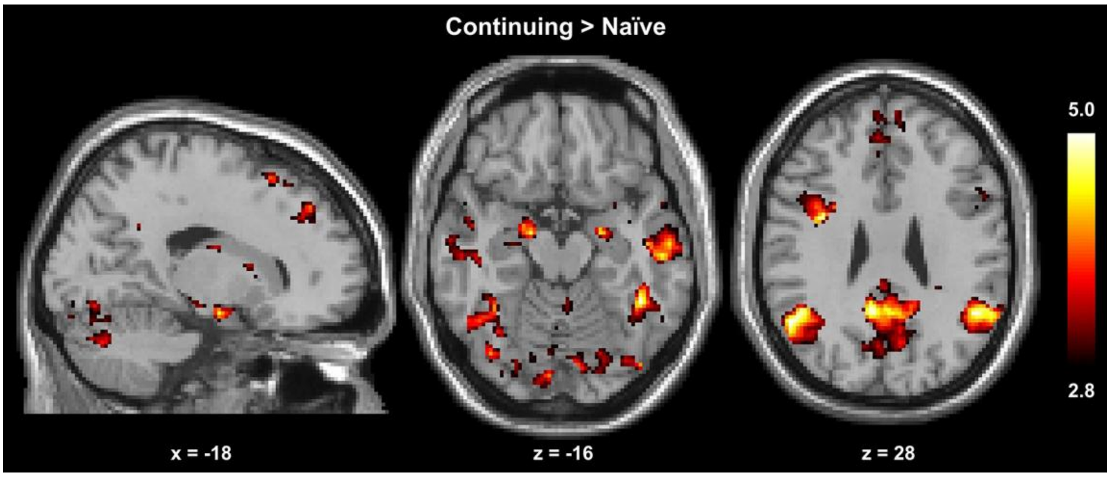

Supplementary Figure 10. GLM results of news clip viewing using permutation test. Brain regions that were more active to the continuing viewing than naïve viewing.

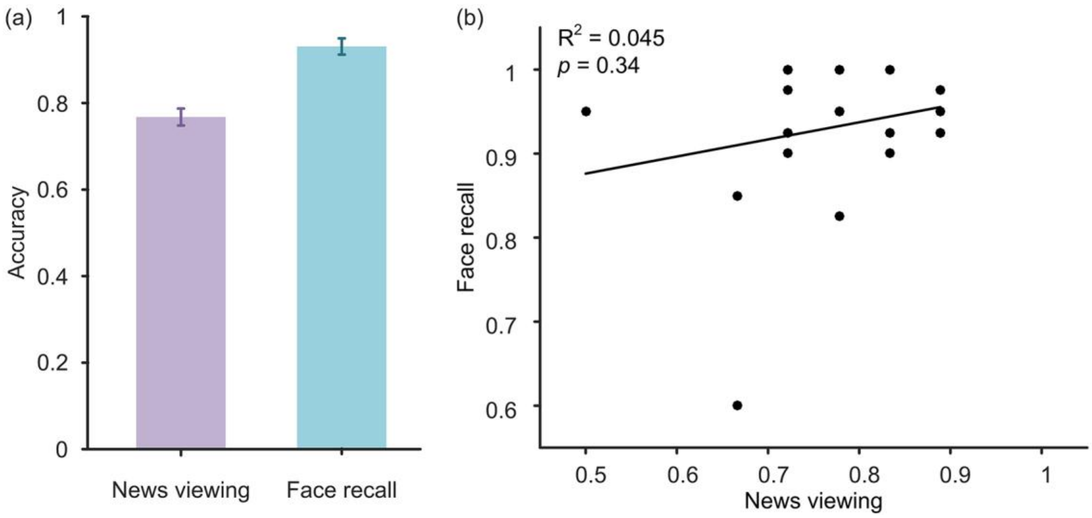

Supplementary Figure 11. (a) The average accuracy of news viewing and face recall paradigms. Error bars signify SEM. (b) Correlation between the accuracy of news viewing and face recall paradigms. Error bars signify SEM.

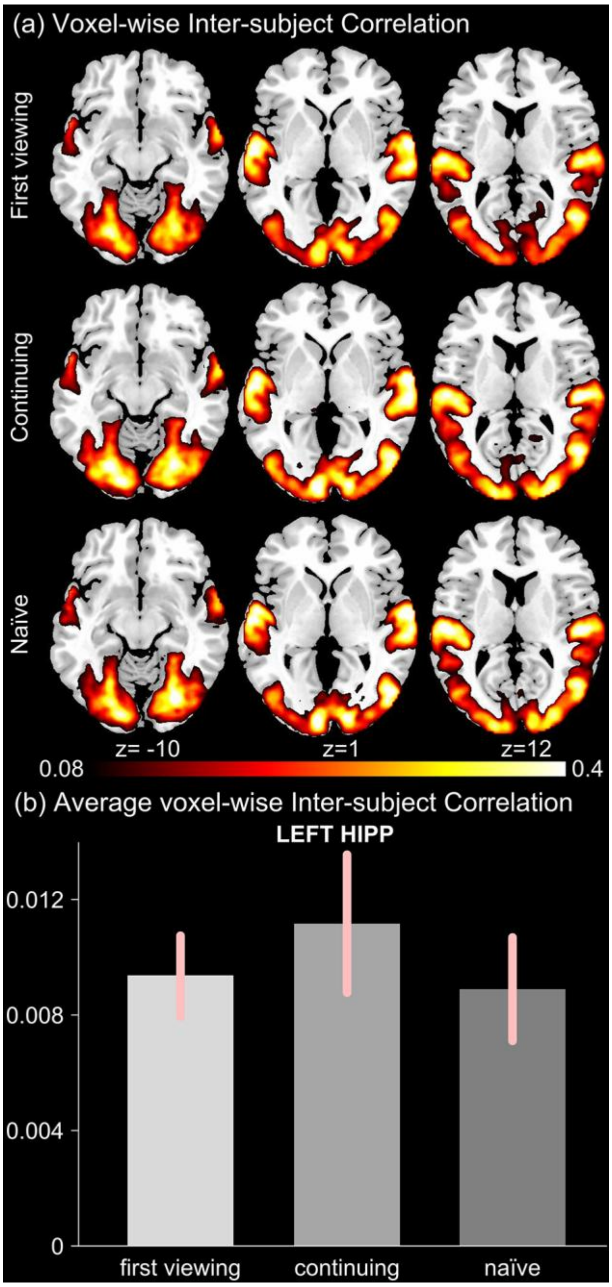

Supplementary Figure 12. Voxel-wise ISC analysis. (a) Group-level ISC maps for the first, continuing and naïve conditions. (b) Average voxel-wise ISC in left hippocampus across news clips under different conditions. Error bars signify standard error of the mean (SEM). HIPP=Hippocampus.

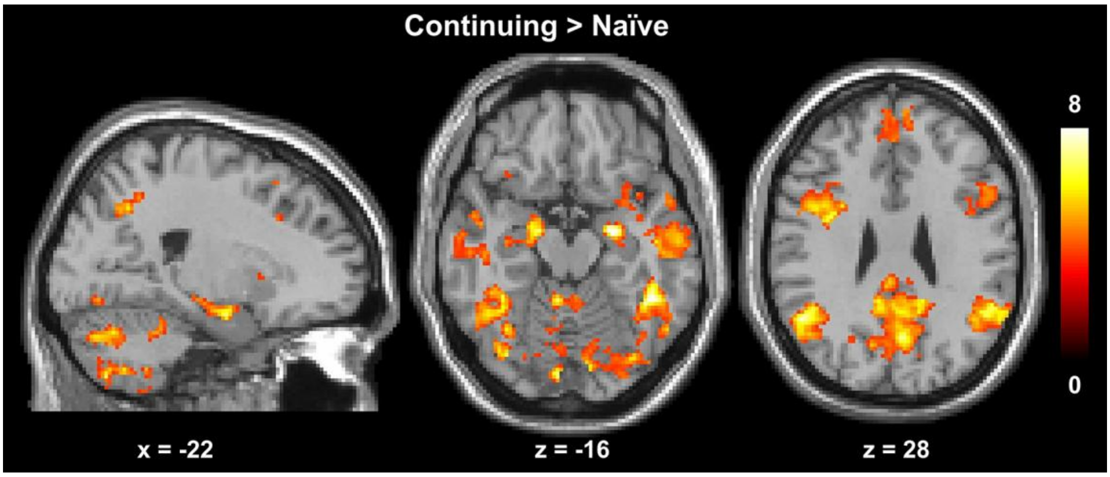

Supplementary Figure 13. GLM results of news clip viewing regarding each condition (all the clips under continuing or naïve condition) as regressor. Brain regions that were more active to the continuing viewing than naïve viewing. Significant voxels were identified using a FWE cluster-corrected threshold $p<0.001$, with a voxel height defined with $p<0.001$.

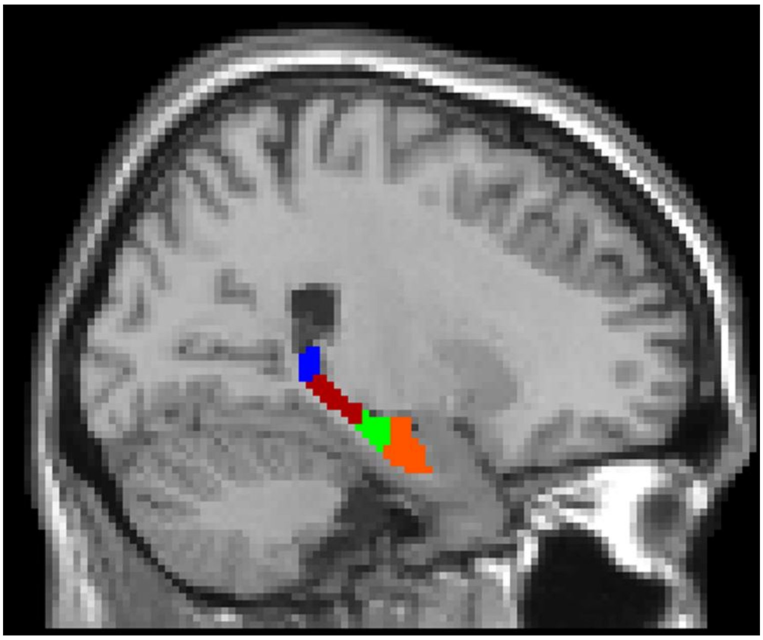

Supplementary Figure 14. Four parcels in left hippocampus. Orange, green, dark red, and dark blue regions (Figure 1, index of 1 to 4) represent parcels from anterior to posterior hippocampus respectively.

Supplementary Table 1. Endogenous connectivity parameters of the winning DCM model 52 (Figure 4).

Connection	Mean	SD
Visual → Precun	0.0157	0.1998
Precun → Visual	0.0771	0.2320
Precun → Angular	-0.0145	0.3073
Precun → Hipp	0.0036	0.2117
Angular → Precun	0.1394	0.1808
Angular → Hipp	0.1334	0.1633
Hipp → Precun	0.1065	0.1600
Hipp → Angular	0.0463	0.2279

Supplementary Table 2. Modulator parameters of the winning DCM model 52 (Figure 4).

Modulator	Mean	SD
Precun → Angular	0.1402	0.6517
Precun → Hipp	0.0496	0.6299
Hipp → Precun	-0.2774	0.6111
Hipp → Angular	0.1062	1.1925

Supplementary Table 3. Audio and video features of news clips under continuing and naïve condition (mean±SEM).

**Features**	**Continuing**	**Naïve**	**Statistics** (p value)
Motion intensity	11.76±0.18	11.59±0.20	0.54
Shot duration	4.91±0.84	4.51±0.47	0.68
Visual excitement	7.65±0.52	8.28±0.44	0.38
Brightness	0.47±0.030	0.49±0.042	0.63
Sharpness	0.92±0.019	0.97±0.012	0.09
Energy	0.0049±0.0020	0.0035±0.0014	0.58
Spectrum entropy	0.89±0.0038	0.89±0.0057	0.93
Silence ratio	0.50±0.030	0.49±0.021	0.67
Zero crossing rate	1827.48±148.71	1922.32±225.81	0.73
mean flux	133.83±4.44	130.01±9.52	0.72

spectral centroid	2724.76±164.31	2991.08±264.64	0.41
fund frequency	173.88±23.08	214.46±46.26	0.44
Saliency	0.98±0.0030	0.98±0.0021	0.45
Angry	0.18±0.020	0.16±0.015	0.67
Fear	0.22±0.027	0.19±0.017	0.40
Happy	0.085±0.017	0.11±0.022	0.47
Neutral	0.11±0.0074	0.12±0.011	0.37
Sad	0.29±0.011	0.32±0.024	0.29
Surprise	0.12±0.017	0.11±0.024	0.92

References

1. Rugg MD, Vilberg KL. Brain networks underlying episodic memory retrieval. Current opinion in neurobiology. 2013;23(2):255-60.
2. Zeidman P, Maguire EA. Anterior hippocampus: the anatomy of perception, imagination and episodic memory. Nature reviews Neuroscience. 2016;17(3):173.
