# Supplementary material for: Effective connectivity of the anterior hippocampus predicts recollection confidence during natural memory retrieval
Source: Nat Commun. 2018 Nov 19;9:4875. doi: 10.1038/s41467-018-07325-4 (PMC6242820; doi:10.1038/s41467-018-07325-4)
Supplement: Supplementary file 2 — Peer Review File [file 41467_2018_7325_MOESM2_ESM.pdf]

This manuscript has been previously reviewed at another journal that is not operating a transparent peer review scheme. The manuscript was considered suitable for publication without further review at Nature Communications
